# Single-Molecule Nanopore Sequencing of the CpG Island from the Promoter of O6-Methylguanine-DNA Methyltransferase Provides Insights into the Mechanism of De Novo Methylation of G/C-Rich Regions

**DOI:** 10.3390/epigenomes9010004

**Published:** 2025-01-26

**Authors:** Alexander V. Sergeev, Daniil P. Malyshev, Adelya I. Genatullina, Galina V. Pavlova, Elizaveta S. Gromova, Maria I. Zvereva

**Affiliations:** 1 Department of Chemistry, M.V. Lomonosov Moscow State University, 119991 Moscow, Russia; daniilmalyshev0@gmail.com (D.P.M.); delyaaa.smile@mail.ru (A.I.G.); gromova@belozersky.msu.ru (E.S.G.); maria.i.zvereva@yandex.ru (M.I.Z.); 2 Institute of Higher Nervous Activity and Neurophysiology of the Russian Academy of Sciences, 117485 Moscow, Russia; lkorochkin@mail.ru; 3 Burdenko National Medical Research Institute for Neurosurgery, 125047 Moscow, Russia

**Keywords:** G-quadruplexes, *MGMT* promoter, DNA methylation, Dnmt3a, nanopore sequencing

## Abstract

Background: The methylation of cytosine residues at CpG sites within the O6-methylguanine-DNA methyltransferase (*MGMT*) promoter is a key biomarker in glioblastoma therapy. The *MGMT* promoter (MGMTp) contains multiple guanine-rich sequences capable of folding into G-quadruplexes (G4s), but their relevance for MGMTp methylation is poorly understood. Objectives: Our study explores the impact of potential G-quadruplex-forming sequences (PQS) in the *MGMT* promoter CpG island on the activity of de novo DNA methyltransferase Dnmt3a. Additionally, we investigate their influence on the accuracy of methylation pattern detection using nanopore sequencing. Methods: Nanopore sequencing was employed to analyze the methylation of 94 clinically significant CpG sites in the human MGMTp using an in vitro de novo methylation system. Circular dichroism spectroscopy was used to identify G4 structures within the MGMTp CpG island. Interactions between the catalytic domain of Dnmt3a and the PQS from the MGMTp were examined by biolayer interferometry. Results: Guanine-rich DNA strands of the PQSs in the MGMTp were hypomethylated, while the complementary cytosine-rich strands were methylated by DNA methyltransferase Dnmt3a with higher efficiency. The accuracy of detecting modified bases in the PQS was significantly lower compared to surrounding sequences. Single-stranded guanine-rich DNA sequences from the MGMTp exhibited strong binding to Dnmt3a-CD, with an affinity approximately 10 times higher than their cytosine-rich complements (*K*_d_ = 3 × 10^−8^ M and 3 × 10^−7^ M, respectively). By binding to Dnmt3a, G4-forming oligonucleotides from MGMTp effectively inhibited the methylation reaction (IC_50_ 6 × 10^−7^ M). Conclusions: The obtained data indicate the role of PQSs in establishing de novo methylation of the *MGMT* promoter. They also highlight the challenges of sequencing guanine-rich regions and the impact of specific de novo methylation patterns on clinical data interpretation.

## 1. Introduction

Brain cancer is among the most aggressive cancer types, with a 5-year survival rate of less than 30% [1]. Gliomas are particularly prevalent within this category, originating from glial cells that support and surround neurons. Glioblastoma multiforme (GBM), the most aggressive glioma subtype, has a five-year survival rate of just 6%. GBM accounts for 45.2% of all malignant central nervous system (CNS) tumors and 80% of primary malignant CNS tumors [2]. These statistics highlight the critical need for molecular biomarkers to enhance tumor classification and inform more effective therapeutic strategies. Well-established molecular biomarkers for CNS tumors include mutations in telomerase reverse transcriptase promoter, tumor suppressor protein 53, isocitrate dehydrogenase 1 and 2, and epidermal growth factor receptor [3]. Additionally, several promising prognostic biomarkers for glioma have been identified in recent years, such as the voltage-gated sodium channel β3 subunit, cyclin-dependent kinase 2, and insulin-like growth factor binding proteins [4,5,6].

Over the past three decades, the primary treatments for high-grade gliomas have remained consistent, comprising maximal surgical resection, external beam radiation therapy, and chemotherapy [2,7]. However, ongoing research is exploring innovative approaches, including gene therapy and immunotherapy. Currently, the standard of care involves the use of temozolomide, an oral cytotoxic DNA-alkylating agent, combined with radiation therapy. Notably, cytosine methylation at CpG sites in the O-6-methylguanine-DNA methyltransferase (*MGMT*) promoter has been shown to significantly improve survival outcomes in patients undergoing this combined treatment.

DNA methylation at CpG sites in mammalian cells is a crucial epigenetic modification that underpins the regulation of gene expression, development, genomic imprinting, and other cellular processes [8,9,10]. DNA methylation has a regulatory role in mammalian development from a zygote into a complex, multicellular adult organism [8]. The cytosine methylation pattern is bimodal: highly methylated intragenic regions with high CpG methylation level coexist with CpG islands—methylation-depleted regions of high CpG density—typically located in the promoters of actively transcribed genes [11,12,13]. This pattern is maintained through the dynamic balance of enzymatic DNA methylation and active or passive demethylation processes [14]. The initial establishment of DNA methylation patterns occurs during early development and is mediated by the de novo DNA methyltransferases (MTases) Dnmt3a and Dnmt3b [15]. Maintenance of these patterns during DNA replication is primarily carried out by Dnmt1.

In mammals, DNA methylation occurs at the C5-position of the cytosine residues, primarily in CpG dinucleotide sequences. All MTases use S-adenosyl-L-methionine (AdoMet) as a methyl group donor. This reaction is catalyzed by the C-terminal catalytic domain of DNMT3A/3B, which form linear tetramers with two active sites in association with the catalytically inactive DNMT3L [15,16]. In addition to its catalytic domain, DNMT3a contains an N-terminal regulatory region composed of PWWP (Pro-Trp-Trp-Pro) [17] and ADD (ATRX-DNMT3-DNMT3L) [18] domains. These domains facilitate specific recruitment of the MTase tetramer to distinct genomic regions by recognizing histone marks, such as methylated (H3K36me2/3 [19]), unmethylated (H3K4me0 [20]), or ubiquitinylated (H2AK119ub [21]) residues [22].

Simultaneously, an increasing body of evidence highlights an additional layer of regulation in MTase activity, mediated by interactions with non-canonical DNA structures, particularly G-quadruplexes (G4s) [23,24,25,26,27,28,29]. G4s are formed through the stacking of G-tetrads—planar arrangements of four guanines stabilized by Hoogsteen hydrogen bonds and centrally located monovalent cations, such as K^+^ or Na^+^ [30,31]. Using the G4-seq technique, over 700,000 G4 structures have been identified in the human genome, with most formations associated with oncogenes, tumor suppressors, and other genes related to cancer development [32]. Additionally, G4-specific antibodies have been utilized to visualize G4 formation within the genomic DNA of human osteosarcoma cells [33], highlighting a potentially important biological role of G4 structures.

G4 structures are classified based on strand polarity: (i) parallel, where all four strands run in the same direction; (ii) antiparallel, where two pairs of strands run in opposite directions; and (iii) hybrid, where three strands run in one direction and the fourth strand runs in the opposite direction [31]. Thus, the activity of MTases in a given genomic region is influenced by a complex integration of signals from histone modifications, the enzyme’s intrinsic sequence specificity [34], protein multimerization [35], and non-canonical DNA structures. This interplay makes it challenging to fully elucidate the mechanisms underlying the de novo establishment of DNA methylation pattern.

Whole-genome sequencing studies of the epigenome have further underscored the role of non-canonical DNA structures in shaping DNA methylation landscapes [27]. A notable correlation has been observed across various tissues: genomic regions enriched in G4 structures tend to exhibit low CpG methylation, whereas regions with high CpG methylation are relatively depleted of G4s. These data were derived from the analysis of 2.1 million CpG sites in humans as part of the Human Epigenome Project. Additionally, the authors experimentally measured methylation at over 600,000 CpG sites across 18 individuals using bisulfite mapping, revealing significantly lower methylation levels within quadruplex-forming regions [27]. These findings suggest that G4 structures act as a genome-wide impediment to CpG site methylation. This observation is particularly significant, given that cytosine methylation plays a critical role in the epigenetic regulation of gene expression in mammals. It should be noted that the role of G4 structures in gene regulatory regions is not exclusively repressive. For instance, a recent study demonstrated that a G4 structure in the MYC proto-oncogene promoter facilitates the recruitment of transcription factors and actively enhances transcription [36]. The specific effect of a G4 structure likely depends on its conformation and its position within the promoter. The coexistence of multiple regulatory mechanisms, such as G4 structures and CpG island methylation, offers enhanced adaptability. If one mechanism fails or operates less efficiently under certain conditions, the other can compensate, ensuring robust gene regulation.

Aberrant methylation patterns are frequently associated with cancer and other diseases [37,38,39], highlighting the importance of understanding the interplay between DNA methyltransferase activity and alternative DNA structures. Recent studies have shown a high density of potential G-quadruplex-forming sequences (PQS) in the promoters of human DNA repair genes [40]. Using various experimental approaches, it has been demonstrated that some of the identified PQSs indeed fold into G4 structures both in vitro and in vivo. Among these, the *MGMT* gene has been studied, which encodes an enzyme responsible for the repair of alkylated guanine residues [41,42]. This enzyme restores damaged (alkylated) guanine by transferring the methyl group from the O6 position of guanine to a cysteine residue in the protein. This mechanism prevents gene mutations, cell death, and oncogenesis caused by alkylating agents [41]. The expression of the *MGMT* gene is primarily regulated by epigenetic modifications, specifically the methylation of the CpG island within the *MGMT* promoter (MGMTp). When the promoter is methylated at CpG sites, the synthesis of the MGMT repair enzyme is significantly reduced, leaving alkylation-induced damage unrepaired. Under these conditions, chemotherapy with alkylating agents targeting cancer cells becomes a viable treatment option. Therefore, determining the methylation status of the *MGMT* promoter is critically important [43].

MGMTp is considered a marker of precancerous lesions and a biomarker for the early diagnosis of various tumors, including gastric cancer, colorectal cancer, breast cancer, squamous cell carcinoma of the oral cavity, and cervical carcinoma [44,45,46,47,48]. It has also acquired a key diagnostic role for brain tumor lesions, serving as a molecular biomarker for selecting anti-cancer therapies [49]. However, several challenges remain for its clinical application, such as achieving consensus on MGMTp methylation detection methods, as these methods vary significantly across laboratories. Additionally, optimal MGMTp methylation thresholds for glioblastoma diagnostics are still lacking [50]. The CpG island in the MGMTp region contains several PQSs, raising the question of their potential influence on Dnmt3a activity, which is responsible for de novo DNA methylation and establishing the MGMTp methylation pattern. Previously, we reported a crosstalk between G4 structures and Dnmt3a-mediated methylation of the *c-MYC* oncogene promoter [26].

In this study, we explored the mechanistic aspects of Dnmt3a function in MGMTp. We focused on the influence of DNA sequence context and non-canonical structures on the methylation process. Using an in vitro methylation model, we examined how G4 structures affect DNA methyltransferase activity. Understanding the mechanisms that shape methylation patterns in MGMTp not only provides a deeper insight into the biology of de novo DNA methylation but also holds the potential to explain variability in clinical data. Additionally, it may enhance the utility of MGMTp methylation as a biomarker for disease prognosis and treatment.

## 2. Results

### 2.1. MGMTp PQS Reduces the Accuracy of Modified Base Identification in Nanopore Sequencing Data

**Figure 1:**
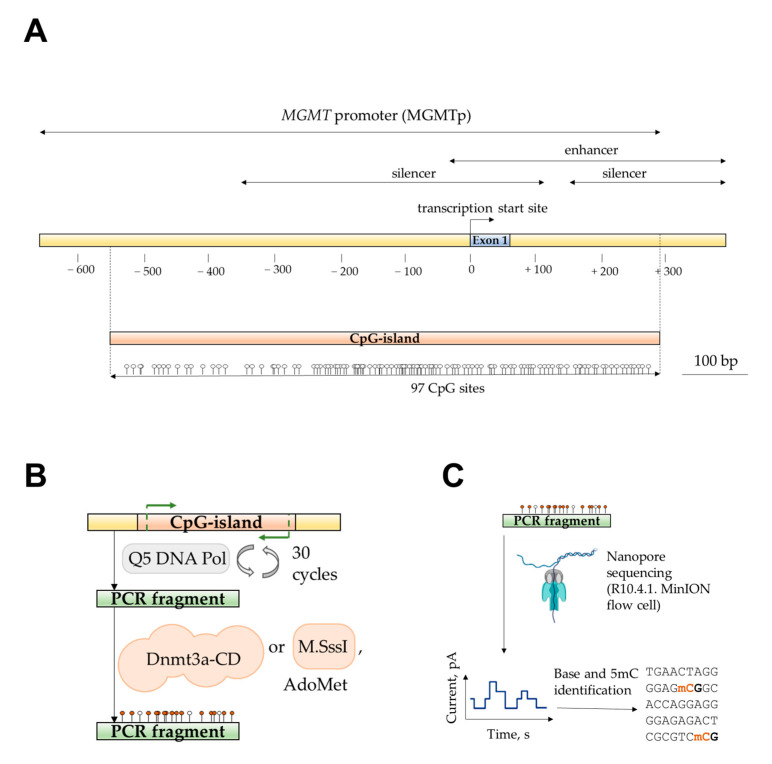
Detection of MGMTp CpG island methylation by nanopore sequencing. (**A**). Schematic representation of MGMTp; the region containing the CpG island is shown. Figure based on [52] and NCBI (Gene ID: 4255). (**B**). PCR amplification and in vitro methylation of a MGMTp region containing CpG sites 3–97 using Dnmt3a-CD or M.SssI. (**C**). Schematic representation of nanopore sequencing of methylated PCR product and detection of 5mC.

In this work, we focused on studying the methylation of a G/C-rich region of MGMTp that contains a CpG island overlapping with the first exon (Figure 1A). The sequence of interest included CpG sites 3–97 out of 97 CpG sites within the island. The 752-base pair (bp) long sequence was amplified from Raji human lymphoblast-like cell line genomic DNA, and the resulting PCR product was referred to as MGMT-752 (Table 1).

Then, we examined the efficiency of de novo methylation of MGMT-752 by the catalytic domain of mouse MTase Dnmt3a (Dnmt3a-CD), which is identical in amino acid sequence to the human enzyme, and is catalytically active in absence of its N-terminal chromatin-targeting part [51]. To this end, MGMT-752 duplex was enzymatically methylated by Dnmt3a-CD in the presence of methyl group donor AdoMet (Figure 1B). For comparison, a portion of MGMT-752 was methylated by a procaryotic monomeric C5 MTase M.SssI from *Spiroplasma* sp. strain MQ1 that targets CpG sites, while the control sample was left unmethylated.

**Figure 2:**
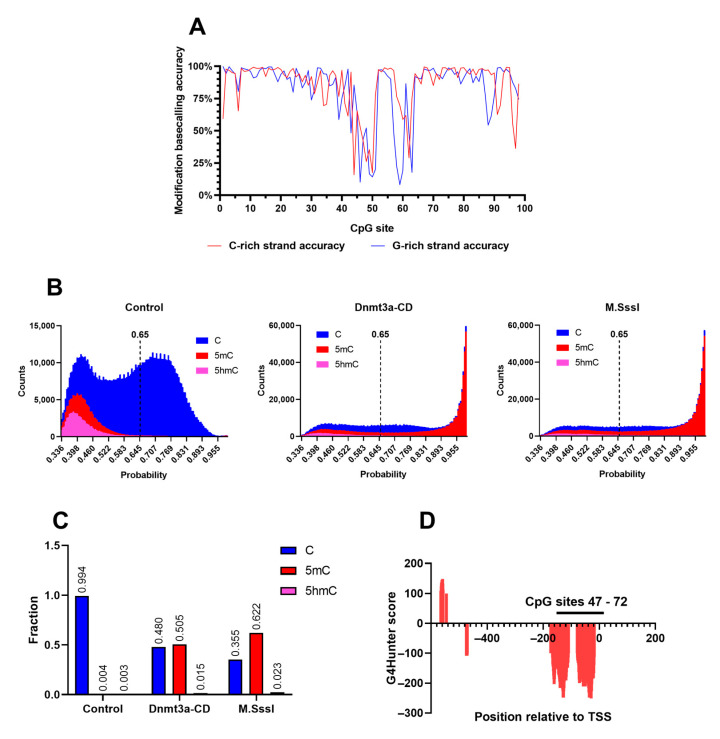
Modification calling of methylated and control PCR fragments of MGMTp CpG island (MGMT-752). (**A**). Distribution of base modification calling accuracy scores. Cytosine- and guanine-rich strands of the PCR fragment (C-rich and G-rich strand, respectively) were analyzed separately. (**B**). Modification probability distribution in unmethylated control and MGMT-752 methylated by Dnmt3a-CD or M.SssI. A confidence threshold of 0.65 (dashed line) was used to filter out erroneous methylation calls. (**C**). Total cytosine modification levels in unmethylated control and MGMT-752 methylated by Dnmt3a-CD or M.SssI. (**D**). PQS coverage of MGMTp CpG island predicted using G4Hunter software. TSS: transcription start site.

In order to determine the enzymatic CpG methylation patterns of the amplicons, we employed nanopore sequencing. Purified DNA samples were analyzed using the Mk1B sequencing device equipped with a MinION Flow Cell (R10.4.1) (Figure 1C). The sequencing run generated 140,670 reads, yielding 117.69 megabases (Mb) of nucleotide data, which were subsequently analyzed using Dorado Basecaller software (version 0.6.2) and mapped to the GRCh38.p14 reference human genome assembly. Here, base modification calling was performed concurrently with basecalling using the supported Dorado model. The analysis utilized a model capable of detecting both 5-methylcytosine (5mC) and 5-hydroxymethylcytosine (5hmC). Although 5hmC was not present in the tested samples, its detection served as a quality control measure for assessing the accuracy of the modification model. Using the unmethylated control amplicon as a reference, the 5mC probability distribution was plotted to evaluate modification calling accuracy across the region of interest. The accuracy distribution, generated with the Modkit Pileup tool, was separately visualized for the C-rich (coding) and G-rich (template) DNA strands of the control amplicon (Figure 2A). Unexpectedly, an anomalous region between CpG sites 40 and 65 exhibited a marked reduction in modification calling accuracy for both DNA strands, with the probability of correctly identifying unmethylated cytosines reaching as low as 25%. In contrast, accuracy across the remainder of the region consistently exceeded 75%, except at the 5′- and 3′- ends of the amplicon, where lower sequencing quality is a well-documented artifact [53].

To quantify the extent of false positive modification calls in the unmethylated sample, the probability distributions for all three cytosine variants—canonical, 5mC, and 5hmC—were plotted for control, Dnmt3a-CD-treated amplicons, and M.SssI-treated amplicons (Figure 2B). A significant amount of low-confidence, erroneous 5mC and 5hmC calls were detected in the control amplicon, likely corresponding to the low accuracy region between CpG sites 47–72. A number of 5hmC calls were also present in the methylated samples in the same low confidence interval. To account for these inaccuracies and exclude the erroneous modification calls, a confidence threshold of 0.65 was used in the following analyses. Next, total amount of modified cytosines was calculated for all three studied amplicons. The 5mC levels were 0.04, 0.505, and 0.622 for control, Dnmt3a-CD-treated amplicons, and M.SssI-treated amplicons, respectively, with all three samples exhibiting a low level (<0.03) of high-confidence 5hmC calls. These calls were considered insignificant in the following analyses, and raising the confidence threshold further would invalidate a large number of correct modification calls. Surprisingly, the anomalous region of low modification calling accuracy closely coincided with the PQSs in the MGMTp CpG island, predicted using G4Hunter^®^ software (Figure 2D). Therefore, the accuracy of detecting modified bases in PQS was significantly reduced compared to the surrounding sequences (Figure 2A,D). Notably, modification calling with the open-source 5mC analysis tool DeepMod 2 demonstrated greater accuracy in detecting canonical cytosines on the control amplicon compared to Oxford Nanopore’s Dorado (Figure S1A).

### 2.2. Differential Methylation of MGMTp DNA Strands by Dnmt3a-CD

**Figure 3:**
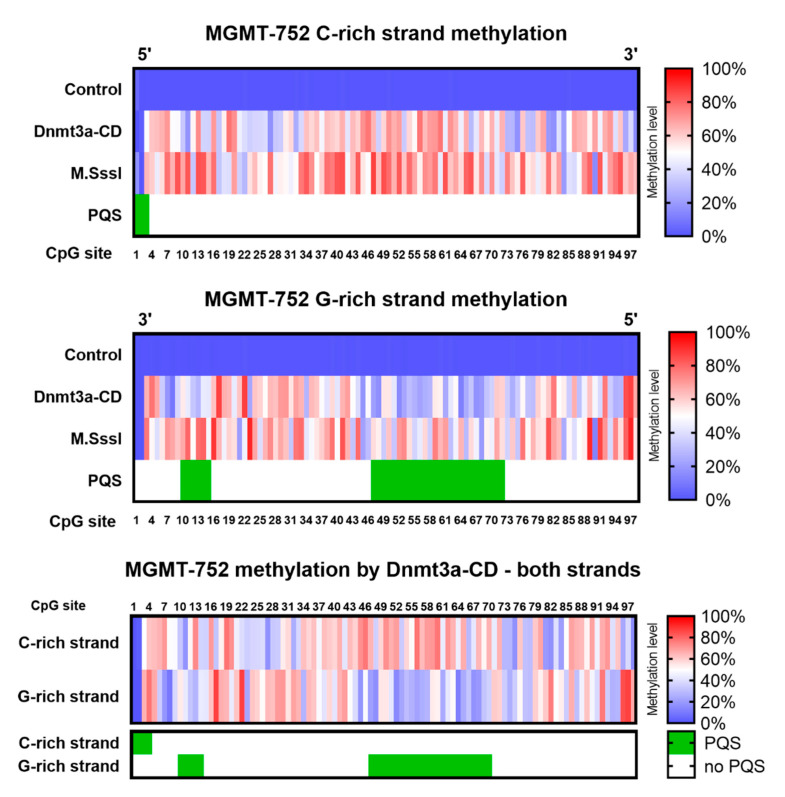
Heatmaps of methylation of MGMT-752 by Dnmt3a-CD or M.SssI. Unmethylated amplicon was used as a negative control. Methylation level represents the probability that the CpG site contains 5mC, averaged from all sequencing reads. **Top panel**: C-strand methylation heatmaps. **Middle panel**: G-strand methylation heatmaps. **Bottom panel**: combined heatmap of C- and G-strand methylation by Dnmt3a-CD. G4 coverage was calculated using G4Hunter software.

The cytosine methylation patterns of the analyzed amplicons were next examined (Figure 3). Single-molecule sequencing allowed to differentiate the methylation patterns of the DNA strands. Control amplicons were uniformly unmethylated, whereas MTase-treated molecules exhibited methylation patterns that varied depending on the DNA strand and the enzyme used.

Unexpectedly, both Dnmt3a-CD and M.SssI exhibited differential methylation patterns between the C- and G-strands of the amplicon, with the C-strand generally displaying higher 5mC levels. Notably, amplicons treated with Dnmt3a-CD revealed a region of pronounced strand-specific methylation differences spanning CpG sites 47 to 72 (Figure 3, bottom panel). Interestingly, this region corresponded to an area previously identified as having reduced modification calling accuracy and overlapped with the PQS in MGMTp, as predicted by G4Hunter software (Figure S2A). Specifically, the G-rich DNA strand in the primary PQS region displayed significantly lower methylation levels compared to its complementary C-rich strand (33% ± 3% vs. 58% ± 3%, respectively; *p* value < 0.0001) (Figure S2B). On the other hand, the difference of methylation efficiency between DNA strands outside of the primary PQS was not significant (47% ± 3% vs. 49% ± 3%, for G-rich and C-rich strands, respectively; *p* value 0.40) (Figure S2C). Additionally, a separate analysis using the DeepMod 2 tool produced a comparable methylation pattern, revealing significantly lower methylation levels in the PQS region of the G-rich strand (Figure S1B). Publicly available datasets from Oxford Nanopore Technologies Open Data were analyzed to validate the methylation calls within the MGMTp region of the GM24385 cell line genomic DNA. The resulting DNA methylation heatmaps demonstrate that methylation calls derived from nanopore data are largely consistent with those obtained through bisulfite sequencing of the same region (Figure S1C).

These findings suggest a possible relationship between the presence of PQS and the regulation of de novo methylation at CpG sites within these sequences. While the PQSs in the MGMT-752 amplicon are unlikely to form stable G4 structures due to competition with the energetically favorable B-form DNA duplex, MGMTp PQSs have been shown to adopt G4 folds in human cells [40]. Mao et al. [25] provided evidence that G4 structures which were observed in human using a G4-specific antibodies influence the methylation at CpG islands. The authors proved the ability of maintenance DNMT1 MTase to colocalize at sites of G4 formation and proposed a mechanism for protecting CpG islands from methylation by G4 structures. Consequently, the next objective of this study was to assess the effect of MGMTp G4 structures on Dnmt3a-CD methylation efficiency and to determine the extent to which these differences could be attributed to the presence of G4s [23] or MTase sequence preferences [34].

### 2.3. MGMTp PQS and G4 Structures Form Stable Complexes with Dnmt3a-CD and Inhibit Its Methylation Activity

**Figure 4:**
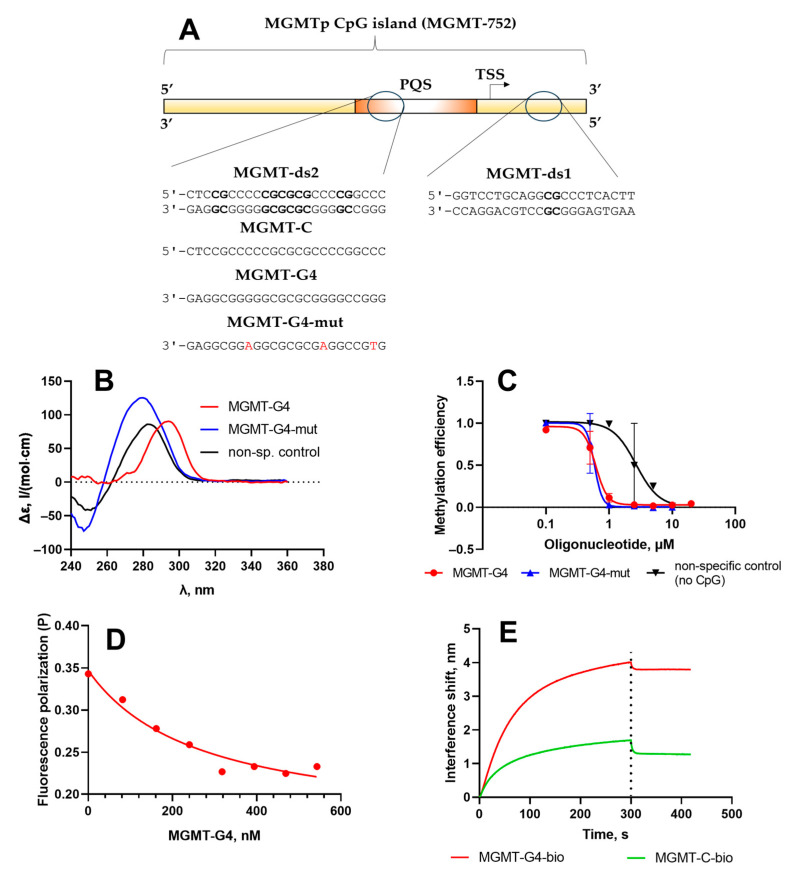
Molecular characterization of the interaction of MGMTp G4 oligonucleotides with Dnmt3a-CD. (**A**). Short oligonucleotides and DNA duplexes based on MGMTp CpG island. (**B**). Circular dichroism studies of MGMT-G4 oligonucleotide and non-G4-forming controls (Table 1). (**C**). Inhibition of methylation of a double-stranded Dnmt3a substrate MGMT-ds1-f by Dnmt3a-CD in the presence of MGMT-G4. Error bars represent the SD from at least two independent experiments. (**D**). Displacement of MGMT-ds1-f from the complex with Dnmt3a-CD in the presence of MGMT-G4 studied by fluorescence polarization. (**E**). Binding of MGMT-G4 to Dnmt3a-CD studied by biolayer interferometry. The experiments were conducted in buffer A.

For this study, we selected a range of short DNA duplexes and oligonucleotides derived from the MGMTp CpG island (Figure 4A). These included a 24-bp double-stranded DNA fragment, MGMT-ds2, from the MGMTp PQS; its G-rich strand, MGMT-G4; and an analog, MGMT-G4-mut, which contained guanine substitutions designed to inhibit G4 formation (Table 1). Additionally, a C-rich oligonucleotide, MGMT-C, and a 24-bp DNA duplex from outside the PQS region (MGMT-ds1) were included. As a reference, a non-specific control oligonucleotide lacking CpG sites (non-sp. control) was also utilized.

The secondary structures of the single-stranded DNA fragments were analyzed using circular dichroism (CD) spectroscopy (Figure 4B). The MGMT-G4 oligonucleotide displayed a positive peak at 295 nm, indicative of a hybrid quadruplex structure characterized by a combination of parallel and antiparallel DNA strands [54]. In contrast, the MGMT-G4-mut oligonucleotide exhibited no evidence of non-canonical structures; its CD spectrum closely resembled that of the single-stranded non-specific control. Earlier, it was shown that a 24-bp oligonucleotide representing the MGMT promoter PQS exhibited CD spectra characteristic of a parallel G4 structure [40]. In contrast, our findings suggest a mixed-hybrid G4 folding conformation for the MGMT-G4 oligonucleotide (Figure 4A). This difference may result from our oligonucleotide covering a slightly different region of the same PQS, incorporating more downstream and fewer upstream nucleotides in the sequence (Figure 4A).

To evaluate the impact of the G4 structure on Dnmt3a-CD function, we investigated the inhibition of the methylation reaction of the fluorescently tagged MGMT-ds1-f duplex by oligonucleotides MGMT-G4 and controls (Table 1), following a previously established methodology (Figure 4C) [26]. MGMT-ds1-f was a 22-bp DNA duplex containing a single central CpG site, derived from a region of the MGMTp CpG island located outside the PQS. Methylation efficiency was measured by protection of methylated DNA from digestion by restriction endonuclease Hin6I. In the absence of MGMT-G4, the MGMT-ds1-f duplex was almost fully methylated by Dnmt3a-CD. However, as the concentration of MGMT-G4 increased, the degree of MGMT-ds1-f methylation decreased, indicating inhibition of Dnmt3a-CD activity (Figure S3). The IC_50_ value, derived from the plot of methylation fraction as a function of MGMT-G4 concentration (Figure 4C), was 0.61 ± 0.03 µM. In contrast, the control oligonucleotide without CpG sites (non-sp. control, Table 1) demonstrated significantly weaker inhibition of Dnmt3a-CD activity (IC_50_ 2.4 ± 0.7 µM). Interestingly, the MGMT-G4-mut, which, according to CD data, lacks a G4 structure, exhibited a similar level of inhibition as MGMT-G4 (IC_50_ 0.59 ± 0.09 µM). In this case, the presence of multiple CpG repeats in MGMT-G4-mut may promote the formation of transient double-stranded structures, resulting in a high affinity for Dnmt3a-CD. Therefore, the G4-forming oligonucleotide MGMT-G4, derived from the MGMTp PQS, effectively inhibited the methylation reaction by tightly binding to Dnmt3a-CD and preventing the MTase from binding to its regular double-stranded substrate.

The mechanism of Dnmt3a–CD interaction with guanine-rich DNA sequences is supported by the results of a DNA displacement experiment involving the Dnmt3a-CD/MGMT-ds1-f complex. Fluorescence polarization demonstrated that adding the unlabeled guanine-rich sequence MGMT-G4, which can form a G4, to the Dnmt3a-CD/MGMT-ds1-f complex decreases the fluorescence polarization signal (Figure 4D). This suggests that the MGMT-G4 oligonucleotide displaces the FAM-labeled DNA duplex from the enzyme’s binding site.

Next, we used bio-layer interferometry (BLI) to investigate the interaction between MGMTp G4 structures or PQS sequences and Dnmt3a-CD and to evaluate their properties as MTase substrates. Biotinylated DNA substrates were immobilized on streptavidin-coated biosensors. The experiment consisted of an association phase, during which the DNA substrates interacted with Dnmt3a-CD, followed by a dissociation phase to observe the stability of the complexes.

The BLI results demonstrate that Dnmt3a-CD binds to MGMT-ds1-bio and MGMT-ds2-bio with high affinity (Figure S4). The dissociation constants (*K*_d_) of the DNA–protein complexes varied by a factor of 4, depending on the number of CpG-sites and the presence of G clusters (Table 2), with the guanine-rich duplex MGMT-ds2-bio binding to Dnmt3a-CD more strongly than the MGMT-ds1-bio duplex. Furthermore, we observed notable differences in the binding affinity of Dnmt3a-CD to complementary cytosine- and guanine-rich DNA strands of MGMT-ds2-bio (Figure 4E). The guanine-rich strand MGMT-G4-bio binds to the protein similarly to G/C-rich MGMT-ds2-bio and is 14 times more strongly than its complementary cytosine-rich strand MGMT-C-bio, confirming the high affinity of Dnmt3a-CD for G4 structures [23]. These findings are supported by the results of a DNA displacement experiment (Figure 4D).

## 3. Discussion

In this study, we characterized, for the first time, the de novo methylation pattern of a long model DNA fragment from MGMTp using a Dnmt3a-CD-based in vitro methylation system (Figure 1). Utilizing nanopore sequencing for the direct detection of methylation products (Figure 2), we identified strand-specific effects and a distinct methylation pattern for Dnmt3a (Figure 3). Specifically, the region of strand-dependent Dnmt3a-mediated methylation coincided with the MGMTp PQS identified by G4Hunter algorithm, leading to highly uneven methylation across the 97 CpG positions of MGMTp.

The observed preferential methylation of the C-rich strand could be attributed to Dnmt3a’s known strong flanking sequence preference [55], favoring CpG sites followed by cytosines and thymines. Bisulfite sequencing experiments have demonstrated that Dnmt3a exhibits preferences for bases flanking the CpG site at more distal positions [56]. These preferences may arise from the influence of the flanking sequence on protein–DNA complex formation or specific steps of the enzymatic reaction. In general, CpG sites flanked by cytosines within three bases in either the 3′ or 5′ direction are expected to be more favorable substrates for Dnmt3a-CD compared to CpG sites flanked by guanines.

Dnmt3a’s distributive mechanism necessitates either dissociation and rebinding to hemimethylated CpG sites to methylate the complementary strand or the binding of a second tetramer to the same site [35]. In contrast, the effects were less pronounced with the monomeric CpG recognizing MTase M.SssI, which in spite of strong homology to ten conserved regions of the tetramerDnmt3a-CD, exhibits a differently arranged complex with DNA [57,58]. Thus, one can suggest that in the context of a PQS with a high density of CpG sites, Dnmt3a tetramers may preferentially methylate CpGs with favorable nucleotide contexts on the C-rich strand, resulting in the accumulation of hemimethylated CpG sites.

The accuracy of 5mC detection within the MGMTp PQS using nanopore sequencing data was significantly lower than for surrounding sequences, underscoring the need for improved modified basecalling models. This limitation, coupled with known challenges in nanopore sequencing of homopolymer regions—such as cytosine and guanine repeats in PQSs—highlights the importance of considering such sequences in the development and refinement of basecalling algorithms. High-GC and homopolymeric sequences are known to be susceptible to basecalling errors, including deletions, insertions, and mismatches [53]. Simultaneously, ONT technology demonstrated a lower overall error rate in non-B DNA motifs compared to Illumina and PacBio, making it particularly advantageous for studying regions such as telomeres and CpG islands [59]. ONT methylation calls have undergone extensive validation in recent years, as evidenced by several studies [60,61], which include the analysis of PQS-containing promoter regions. Notably, nanopore methylation calls demonstrated a strong correlation with Sanger bisulfite sequencing data at the TRPA1 promoter, showing discrepancies primarily in the methylation range below 20%. Moreover, a recent study specifically validated ONT methylation calls for four CpGs within the MGMT promoter [62] using pyrosequencing, revealing a significant correlation between the results. Our findings indicate that the distinctive sequence composition of the MGMTp CpG island, characterized by clusters of homopolymeric cytosine and guanine tracts, introduces a different type of error—an increase in low-confidence erroneous methylation and hydroxymethylation calls.

The alignment of strand-specific methylation sites with PQS regions (Figure 3) suggested an influence of these structures on methylation in human cells. G4 structures might form locally in the G-rich strand, potentially affecting the catalytic activity of Dnmt3a-CD (Figure 4). The differences in the dissociation constants of Dnmt3a-CD complexes with MGMTp DNA duplexes and oligonucleotides were also revealed (Table 2). One possible explanation is that G4 structures, formed locally on the G-rich strand, may provide an independent interaction site for DNMT3a-CD, attracting the enzyme to G4s and resulting in hypomethylation of the surrounding regions. This result is consistent with the high binding affinity of recombinant human DNMT3A to G4 containing oligonucleotides derived from promoters of various human genes (*CDKN1C*, *c-MYC*, and others) [23]. At the same time, if the double-stranded form is preserved, increased methylation can be expected in PQS.

The characteristic methylation pattern observed in brain tumor samples aligns with our findings. This pattern, as reported by Shah et al. [63] in their comprehensive analysis of MGMT promoter methylation, correlates with MGMT mRNA expression and patient responses to primary GBM therapy. Our data may provide insight into the specific G4-forming sequence of the MGMT promoter that is predictive of gene silencing and clinical response.

The ability of PQS from MGMTp to fold into G4 structures upon interaction with G4-specific ligands in human glioblastoma cells was demonstrated by Fleming et al. in 2018 [40]. This finding was supported by bioinformatic analysis and circular dichroism (CD) experiments. However, these results and our CD experiments using short DNA oligonucleotides may not directly reflect the precise topology of MGMT promoter PQS G4 structures within human cells. Instead, they provide evidence supporting the potential for G4 formation in the studied region. Further studies are needed to clarify the interplay between strong flanking sequence preference and interaction with histone and non-canonical structures in de novo methylation of MGMTp CpG island by Dnmt3a.

Our findings also necessitate a reevaluation of research data obtained using methods such as the OneStep qMethyl™ Kit from Zymo Research [64]. This kit detects locus-specific DNA methylation by selectively amplifying methylated regions of DNA and comparing them to human non-methylated DNA. The reference DNA is purified from cells with genetic knockouts of both DNMT1 and DNMT3b DNA methyltransferases, resulting in a low level of DNA methylation (~5%) [65]. In the context of MGMTp, the application of this kit must be reconsidered, as the presence of PQS could influence the interpretation of methylation levels and compromise the accuracy of the results.

## 4. Materials and Methods

**Oligonucleotides** (Table 1) were commercial products (Genterra, Moscow, Russia). Some of the oligonucleotides contained biotin or the fluorescent label 6-carboxyfluorescein (FAM). The concentrations of the oligonucleotides were determined spectrophotometrically. DNA duplexes and G4 structures were formed using by heating at 95 °C for 3 min and slow cooling to 4 °C in buffer A: 20 mM HEPES-NaOH (pH 7.5), 100 mM KCl, 1 mM EDTA, 1 mM 1,4-dithiothreitol.

**MGMTp amplification.** MGMTp CpG island PCR product (MGMT-752; Table 1) was amplified using 1 µg of genomic DNA from the Raji human lymphoblast-like cell line (Eurogen, Moscow, Russia) as the template, along with NEB Q5^®^ High-Fidelity DNA Polymerase (New England Biolabs, MA, USA) and the primers 5′-GGGATTCTCACTAAGCGGGC and 5′-CTGGCACCTAGAGGTAAGGC. The thermocycling conditions were as follows: initial denaturation at 98 °C for 30 s, followed by 30 cycles of denaturation at 98 °C for 5 s, primer annealing at 72 °C for 10 s, and extension at 72 °C for 30 s. A final extension step was performed at 72 °C for 2 min. The PCR products were analyzed by 1% agarose gel electrophoresis, and bands corresponding to the 752-bp product were excised and purified using the Cleanup S-Cap DNA Purification Kit (Eurogen, Moscow, Russia) following the manufacturer’s instructions.

**Enzymes.** To obtain Dnmt3a-CD, Escherichia coli BL21 (DE3) cells were transformed with plasmid pET-28a(+) carrying the gene encoding Dnmt3a-CD with an N-terminal 6 × His tag. Subsequently, Dnmt3a-CD was isolated and purified using metal-affinity chromatography on Co^2+^-containing TALON^®^ resin (GE Healthcare, Chicago, IL, USA). Plasmid pET-28a(+) encoding Dnmt3a-CD was provided by Prof. A. Jeltsch (University of Stuttgart, Stuttgart, Germany). R.Hin6I and M.SssI were commercial products (SibEnzyme, Novosibirsk, Russia). The purity of the protein samples was evaluated using electrophoresis in a 12% SDS-polyacrylamide gel. The protein concentrations per protein monomer were determined using the Bradford assay. The proteins were stored at −80 °C.

**DNA methylation.** 300 ng of the MGMT-752 amplicons were methylated using either Dnmt3a-CD (2 µM) or M.SssI (5 U) in the presence of AdoMet (25 µM) (Sigma, Steinheim, Germany) for 1 h at 37 °C in buffer A. The methylated amplicons were purified using the Cleanup S-Cap DNA Purification Kit.

**Sequencing Library Preparation and Nanopore Sequencing.** Sequencing libraries were prepared using 130 ng of either Dnmt3a-CD/M.SssI-treated, or unmethylated MGMT-752 amplicons following the Ligation Sequencing Amplicons—Native Barcoding Kit 24 V14 (SQK-NBD114.24) protocol (Oxford Nanopore, Oxford, UK). The sequencing mix was prepared with 30 µl of the barcoded DNA library. The mix was loaded onto a MinION R10.4.1 flow cell and run on an Mk1-B sequencing unit (Oxford Nanopore, Oxford, UK). The sequencing run lasted 1 h and 56 min, yielding 141,000 raw reads and 177 Mb of sequence data in POD5 format. The run was monitored using MinKNOW software (version 5.7.2). Offline basecalling of raw POD5 data was performed with the Dorado Basecaller tool (version 0.6.2) [66] using the supported dna_r10.4.1_e8.2_400bps_sup@v5.0.0 model. The following parameters were used: –modified-bases 5mCG_5hmCG; --kit-name SQK-NBD114-24. The resulting .BAM files containing basecalled reads with base modification calls were mapped to the GRCh38.p14 reference human genome assembly using Dorado aligner tool. DNA methylation levels and profiles were then analyzed using the Modkit Pileup tool (version 0.2.7) [67]. The following parameters were used: --motif CG 0; --with-header; --ref NC_000010.11\[129465781..129468355\].fa. In methylation calling accuracy analysis, ‘--no-filtering’ parameter was included. In methylation pattern analysis, parameters ‘--filter-threshold 0.65 --mod-thresholds m:0.65 --mod-thresholds h:0.65′ were used instead.

The methylation levels were visualized with Prism GraphPad software (version 8.0.1). The modification calling accuracy was defined as a probability that the cytosine is correctly identified as unmethylated. Therefore, an accuracy of 100% represented a 100% average probability among all sequencing reads that the position contained an unmodified cytosine, while an accuracy of 0% meant that there were equal probabilities of cytosine, 5mC, and 5hmC being present in the analyzed CpG site.

For comparison, 5mC calls in the unmethylated MGMT-752 amplicon were generated using DeepMod2 tool (version 0.3.0) [68]. Here, base modification calling was conducted after basecalling by Dorado Basecaller. The following parameters were used with DeepMod2 Detect command: --bam eb99195cdaae85326c878417bf262ed568a6d746_SQK-NBD114-24_barcode05.bam; --input pod5/; --model bilstm_r10.4.1_5khz_v4.3; --file_type pod5; --ref NC_000010.11\[129465781..129468355\].fa; --output deepmod2/--threads 8 --seq_type dna. The input .BAM file for DeepMod2 containing basecalled reads without base modification information was generated using Dorado Basecaller tool as detailed above, excluding the –modified-bases parameter. DNA methylation profiles were analyzed with the Modkit Pileup tool as described above. Methylation accuracy distributions were plotted with Prism GraphPad software (version 8.0.1).

Datasets used to validate 5mC calls were sourced from the Oxford Nanopore Technologies Open Data repository, available on Amazon Web Services S3 (https://labs.epi2me.io/dataindex/, accessed on 2 January 2025). BED tables containing methylation frequencies, derived from either bisulfite sequencing or nanopore sequencing of GM24385 cell line genomic DNA, were used to visualize and compare the methylation patterns in the MGMTp region.

**Statistical Analysis.** The significance of the observed effects was assessed using a two-sided *t*-test with unequal variances, performed in Prism GraphPad software (version 8.0.1). Methylation efficiencies within or outside the primary PQS region of MGMTp (CpG sites 47–72) were compared between the C-rich and G-rich DNA strands, and two-tailed *p* values were calculated. Results were reported as mean ± SEM. PQS regions were identified with G4Hunter web application (https://bioinformatics.ibp.cz/#/analyse/quadruplex, accessed on 2 January 2025).

**CD Measurements.** CD spectra of oligonucleotides were recorded in a quartz cuvette of 10 mm optical path length at room temperature in buffer A on a Chirascan CD spectrometer (Applied Photophysics Ltd., Surrey, UK) equipped with a thermoelectric controller. The DNA concentration (~2 μM concentration per oligonucleotide strand) was chosen to attain an absorption of 0.6–0.8 at 260 nm, which gives an optimum signal-to-noise ratio. The measurements were performed in the 230–350 nm wavelength range at a scanning speed of 30 nm/min and a signal averaging time of 2 s with a constant flow of dry nitrogen. The CD spectra were normalized to molar circular dichroism (Δε) using molar strand concentration as a reference. Spectra were baseline-corrected for signal contributions caused by the buffer and processed with GraphPad Prism 8.0.1 (GraphPad Software, San Diego, CA, USA).

**Inhibition of DNA methylation by G4-forming oligonucleotides.** MGMT-ds1-f (300 nM) was methylated by 2 μM Dnmt3a-CD in the presence of various concentrations of MGMT-G4, MGMT G4-mut or non-specific control oligonucleotide for 1.5 h at 37 °C in buffer A containing AdoMet (25 μM). Methylation efficiency was analyzed by the protection of methylated DNA from cleavage by R.Hin6I (G↓CGC). After digestion with R.Hin6I, the mixtures were analyzed on 20% polyacrylamide gel containing 7 M urea, with the determination of the extent of methylation as described in [26]. The gels were visualized using a Typhoon FLA 9500 scanner (GE Healthcare Life Sciences, Buckinghamshire, UK), and the fluorescence intensities of intact DNA and cleavage products were determined. The methylation efficiency was calculated using GelQuantNET version 1.7.8. using the equation:

$$Methylation\;efficiency=\frac{w_{0}-w_{Dnmt3a}}{w_{0}}$$

where *w*_0_ is the extent of DNA cleavage before methylation and *w*_*Dnmt*3*a*_ is the extent of DNA cleavage after methylation by Dnmt3a-CD. IC_50_ values were calculated via fitting the dependence of the extent of methylation on the concentration of an oligonucleotide using GraphPad Prism 8.0.1 (GraphPad Software, San Diego, CA, USA).

**Bio-layer interferometry.** DNA duplexes or oligonucleotides (690 nM) were used for the experiment. The binding buffer contained 20 mM HEPES-NaOH (pH 7.5), 100 mM KCl, 1 mM EDTA, 0.2 mM DTT, 100 µM AdoHcy, 5% glycerol, 0.02% Tween-20, and 0.5 mg/mL BSA. Bio-layer interferometry (BLI) analyses were performed using the BLItz instrument (ForteBio, Fremont, CA, USA) in extended kinetics mode with stirring at 2200 rpm in triplicate. Streptavidin biosensors (ForteBio, Fremont, CA, USA) were hydrated in the binding buffer for 10 min. prior to measurements. The optimized BLI protocol included the following steps: (i) incubation for 30 s; (ii) biotinylated DNA substrate immobilization for 120 s; (iii) sensor wash for 30 s; (iv) DNA binding to Dnmt3a-CD (690 nM in binding buffer) for 300 s; (v) dissociation of the DNA-protein complex in binding buffer for 120 s. All measurements were conducted in black microtubes (Sigma-Aldrich, New York, NY, USA) using a minimum of 300 µL of the appropriate solution. The resulting binding curves were fitted to a 1:1 DNA:protein binding model using an exponential approximation using GraphPad Prism 8.0.1 (GraphPad Software, San Diego, CA, USA).

## 5. Conclusions

We demonstrated that PQS within the MGMT promoter influence the activity of MTase Dnmt3a, a key enzyme responsible for de novo methylation and establishing the MGMT promoter’s methylation pattern. Our findings underscore the role of PQS in regulating MGMT promoter methylation and highlight the technical challenges posed by guanine-rich sequences in nanopore sequencing. Specifically, the presence of PQS in the sequenced region impacts basecalling accuracy in nanopore data, emphasizing the need to account for such structures during analysis. We found the strong specific binding of Dnmt3a-CD to the G4-forming oligonucleotide MGMT-G4, derived from the MGMTp PQS, as well as inhibition of Dnmt3a-CD activity by this oligonucleotide. Also, effective binding of guanine-rich duplex MGMT-ds2 to Dnmt3a-CD was observed. These findings suggest that Dnmt3a-CD recognizes MGMTp CpG island G4 structures and competes with the DNA duplex substrate, thereby modulating the methylation efficiency at nearby CpG sites.

## Figures and Tables

**Table 1:** MGMTp DNA duplexes and oligonucleotides that are used in this study.

Description	Name	Sequence *
MGMT promoter CpG island amplicon	MGMT-752	5′-TGACTAGGGGAG**CG**GCACCAGGAGGGGAGAGACT**CGCG** CTC**CG**GGCTCAG**CG**TAGC**CG**CCC**CG**AGCAGGAC**CG**GGATTC TCACTAAG**CG**GG**CG**C**CG**TCCTA**CG**ACCCC**CGCGCG**CTTTCA GGACCACT**CG**GGCA**CG**TGGCAGGT**CG**CTTGCA**CG**CC**CGCG**G ACTATCCCTGTGACAGGAAAAGGTA**CG**GGCCATTTGGCAAA CTAAGGCACAGAGCCTCAGG**CG**GAAGCTGGGAAGG**CG**C**CG**C C**CG**GCTTGTAC**CG**GC**CG**AAGGGCCATC**CG**GGTCAGG**CG**CAC AGGGCAG**CG**G**CG**CTGC**CG**GAGGACCAGGGC**CG**G**CG**TGC**CG**G **CG**TCCAG**CG**AGGATG**CG**CAGACTGCCTCAGGCC**CG**G**CG**C**CG** C**CG**CACAGGGCATG**CG**C**CG**ACC**CG**GT**CG**GG**CG**GGAACACCC **CG**CCCCTCC**CG**GGCTC**CG**CCCCAGCTC**CG**CCCC**CGCGCG**CC C**CG**GCCC**CG**CCCC**CGCGCG**CTCTCTTGCTTTTCTCAGGTCC T**CG**GCTC**CG**CCC**CG**CTCTAGACCC**CG**CCCCA**CG**C**CG**CCATC CC**CG**TGCCCCT**CG**GCCC**CG**CCCC**CGCG**CCC**CG**GATATGCTG GGACAGCC**CGCG**CCCCTAG AA**CG**CTTTG**CG**TCC**CG**ACGCC**C** **G**CAGGTCCT**CGCG**GTG**CG**CAC**CG**TTTG**CG**ACTTG GTGAGTG TCTGGGT**CG**CCT**CG**CTCC**CG**GAAGAGTG**CG**GAGCTCTCCCT **CG**GGA**CG**GTGGCAGCCT**CG**AGTGGTCCTGCAGG**CG**CCCTCA CTT**CG**C**CG**T**CG**GGTGTG
G4-forming oligonucleotides	MGMT-G4	5′-GGGC**CG**GGG**CGCGCG**GGGG**CG**GAG
	MGMT-G4-mut	5′-G T GC**CG**G A G**CGCGCG**G A GG**CG**GAG
	MGMT-G4-bio	5′-bio-GGGC**CG**GGG**CGCGCG**GGGG**CG**GAG
Controls	MGMT-C	5′-CTC**CG**CCCC**CGCGCG**CCC**CG**GCCC
	MGMT-C-bio	5′-bio-CTC**CG**CCCC**CGCGCG**CCC**CG**GCCC
	non-specific control (no CpG)	5′-CTGAATACTACTTCCTACCCCTTACCTGAT
DNA duplexes	MGMT-ds1-f	5′-FAM-GGTCCTGCAGG**CG**CCCTCACTT 3′-CCAGGACGTCC**GC**GGGAGTGAA
	MGMT-ds1-bio	5′-bio-GGTCCTGCAGG**CG**CCCTCACTT 3′-CCAGGACGTCC**GC**GGGAGTGAA
	MGMT-ds2-bio	5′-bio-CTC**CG**CCCC**CGCGCG**CCC**CG**GCCC 3′-GAG**GC**GGGG**GCGCGC**GGG**GC**CGGG

* In the case of MGMT-752, only the sequence of the cytosine-rich (coding) strand is shown. bio—biotin, f—5-carboxyfluorescein. MGMTp PQSs are highlighted in gray. Exon 1 sequence is highlighted in black. CpG sites are shown in bold; G4-disrupting substitutions are highlighted in red.

**Table 2:** Dissociation constants of Dnmt3a-CD complexes with MGMTp DNA duplexes and oligonucleotides determined using BLI.

DNA Substrate	*K*_d_, nM
MGMT-ds1-bio	65 ± 2
MGMT-ds2-bio	15 ± 0.5
MGMT-G4-bio	14 ± 0.4
MGMT-C-bio	211 ± 21

## Data Availability

The raw data supporting the conclusions of this article will be made available by the authors on request.

## Supplementary Materials

The following supporting information can be downloaded at: https://www.mdpi.com/article/10.3390/epigenomes9010004/s1, Figure S1: Distribution of base modification calling accuracy scores of unmethylated control amplicon MGMT-752 generated using DeepMod2 software. Figure S2: Methylation efficiencies of MGMT-752 by Dnmt3a-CD calculated from nanopore sequencing data. Figure S3: Inhibition of methylation of a DNA duplex MGMT-ds1-f by MGMT-G4 oligonucleotide; Figure S4: Binding of MGMT-G4 to Dnmt3a-CD studied by biolayer interferometry.
