# Supplementary material for: Single-Molecule Nanopore Sequencing of the CpG Island from the Promoter of O6-Methylguanine-DNA Methyltransferase Provides Insights into the Mechanism of De Novo Methylation of G/C-Rich Regions
Source: Epigenomes. 2025 Jan 26;9(1):4. doi: 10.3390/epigenomes9010004 (PMC11843895; doi:10.3390/epigenomes9010004)
Supplement: Supplementary file 1 [file epigenomes-09-00004-s001.zip › epigenomes-3380002-supplementary.pdf]

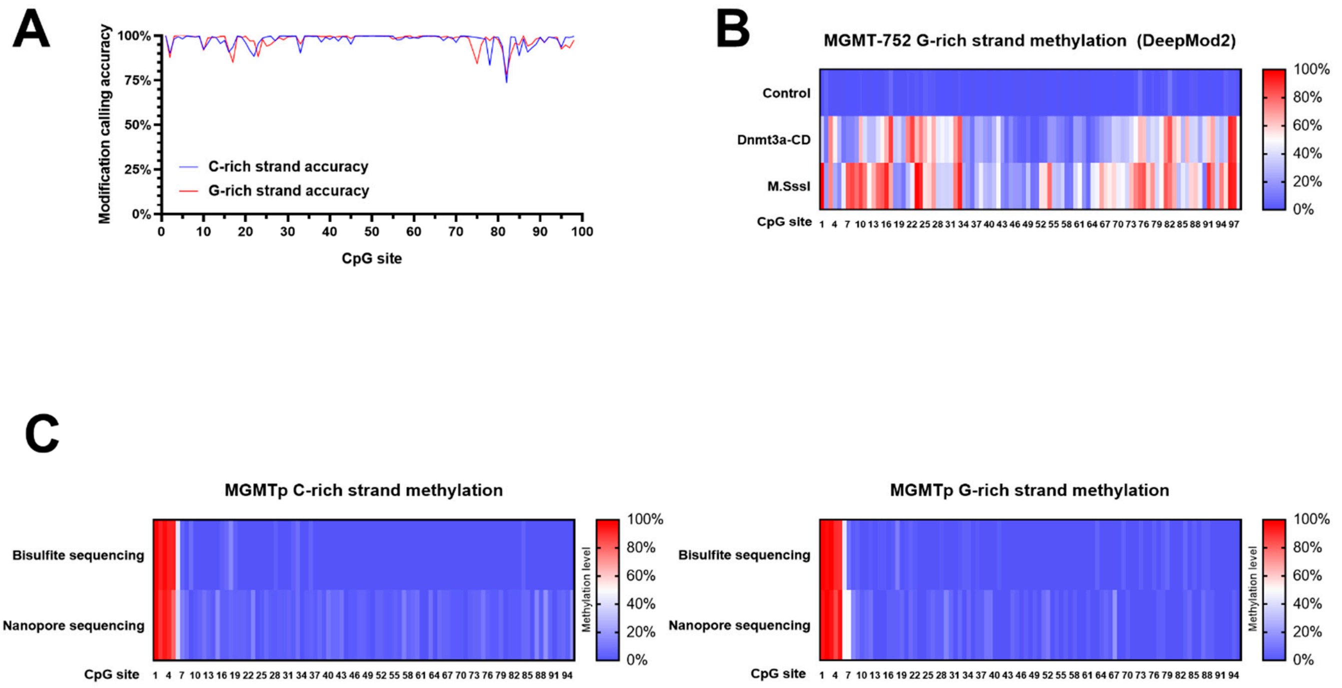

Figure S1. Validation of MGMTp methylation data obtained with nanopore sequencing. A. Distribution of base modification calling accuracy scores of unmethylated control amplicon MGMT-752 generated using DeepMod2 software. Cytosine- and guanine-rich strands of the amplicon (C-rich and G-rich strand, respectively) were analyzed separately. B. Heatmaps of methylation of C-rich strand of MGMT-752 by Dnmt3a-CD or M.SssI generated by DeepMod2. C. Comparison of MGMTp methylation levels in GM24385 cell line between bisulfite and nanopore sequencing. Datasets: Oxford Nanopore Technologies Open Data (https://labs.epi2me.io/dataindex/).

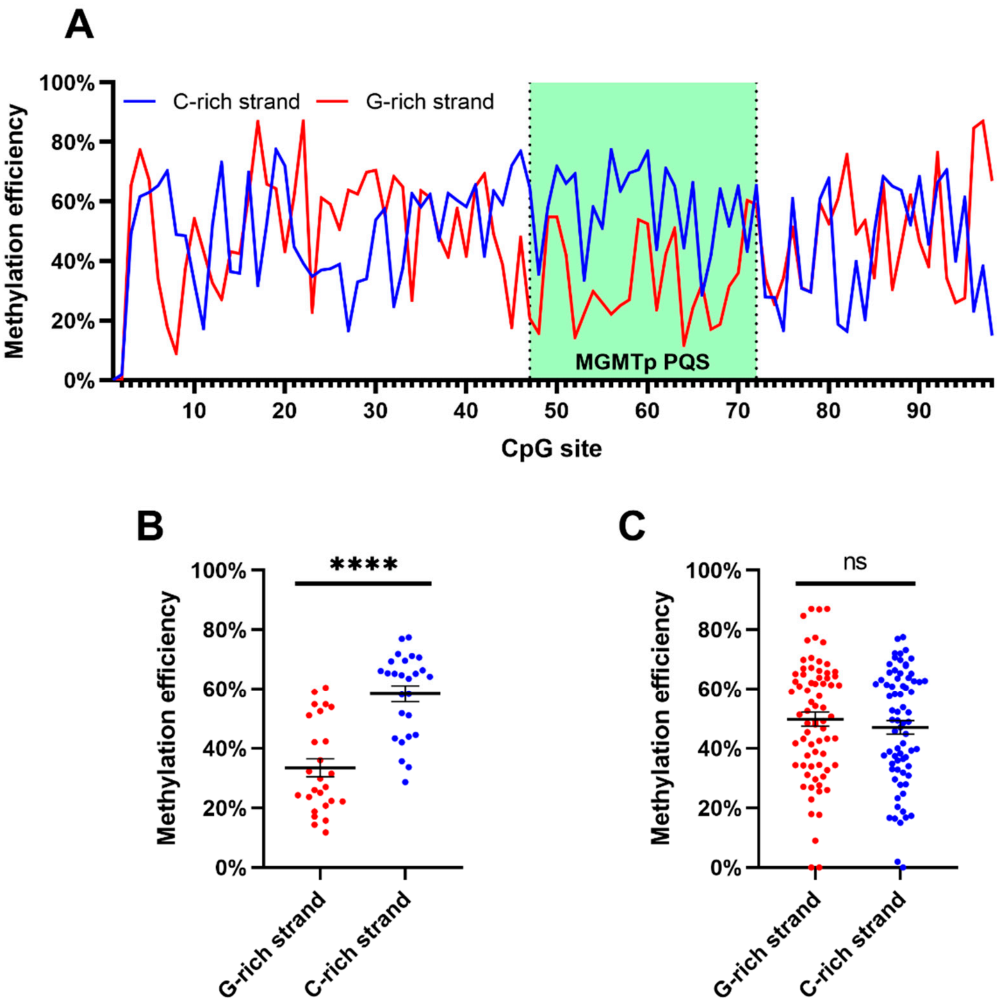

Figure S2. Methylation efficiencies of MGMT-752 by Dnmt3a-CD calculated from nanopore sequencing data. A. Distribution of methylation efficiency values across MGMT-752 following methylation by Dnmt3a-CD, with the primary MGMTp PQS region highlighted. B. Methylation efficiencies at individual CpG sites within the primary MGMTp PQS. C. Methylation efficiencies at individual CpG sites outside the primary MGMTp PQS. Horizontal lines and error bars represent the mean ± SEM.

Dnmt3a-CD	-	-	+	+	+	+	+	+	+	+
R. Hin6I	-	+	+	+	+	+	+	+	+	+
MGMT-G4, µM	0	0	0	0,1	0,5	1	2,5	5	10	20

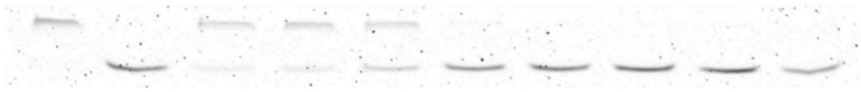

Figure S3. Inhibition of methylation of a DNA duplex MGMT-ds1-f by MGMT-G4 oligonucleotide. The products of MGMT-ds1-f cleavage induced by the R.Hin6I after its methylation with 2 µM Dnmt3a-CD in buffer A in the presence of 25 µM AdoMet and MGMT-G4 (MGMT-G4 concentrations indicated above the gel lanes) were analyzed on 20% polyacrylamide gel containing 7M urea.

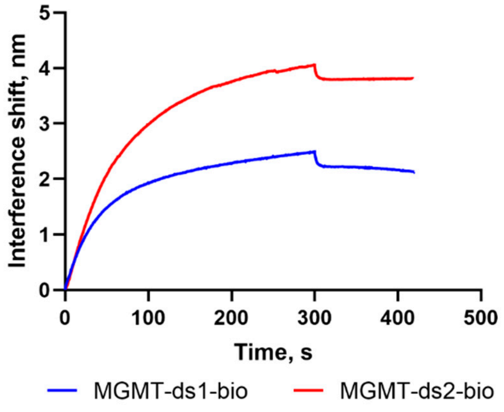

Figure S4. Binding of MGMT-G4 to Dnmt3a-CD studied by biolayer interferometry. The experiments were conducted in a HEPES-NaOH (pH 7.5) solution containing 100 mM KCl at 25°C.
